# Analgesic and Neurorestorative Effects of αO-Conotoxin GeXIVA[1,2] in Diabetic Neuropathic Pain and Postherpetic Neuralgia

**DOI:** 10.3390/toxins18060249

**Published:** 2026-05-29

**Authors:** Rongyan He, Shuting Xiao, Xiaoying Liang, Qiuyu Cao, Shaoxian Wu, Sulan Luo

**Affiliations:** Guangxi Key Laboratory of Special Biomedicine, School of Medicine, Guangxi University, Nanning 530004, China; 2328391048@st.gxu.edu.cn (S.X.); liangcai52711@163.com (X.L.); autumnrain1999@163.com (Q.C.); wsx14050@163.com (S.W.)

**Keywords:** α-conotoxin GeXIVA[1,2], chronic neuropathic pain, inflammatory cytokines, cell infiltration, neural structures

## Abstract

Chronic neuropathic pain, particularly diabetic neuropathic pain and postherpetic neuralgia, severely impairs patients’ quality of life due to their complex mechanisms and recurrent, long-term nature, making treatment challenging. This study aimed to evaluate the analgesic efficacy of α-conotoxin GeXIVA[1,2], a selective antagonist of the α9α10 nicotinic acetylcholine receptor (nAChR), in rat models of diabetic neuropathic pain and postherpetic neuralgia and investigate its associated physiological and pathological effects. GeXIVA[1,2] was administered continuously for three weeks, with mechanical hypersensitivity assessed through pain sensitivity tests, and behavioral assessments conducted to examine motor coordination and gait. Additionally, neural tissue structure and inflammation were analyzed. The results demonstrated that GeXIVA[1,2] significantly alleviated mechanical hypersensitivity in both diabetic neuropathic pain and postherpetic neuralgia models, with greater efficacy than gabapentin and no signs of tolerance. Behavioral tests indicated no significant effects on motor coordination or gait. Further analysis revealed that GeXIVA[1,2] reduced pro-inflammatory cytokine levels, decreased immune cell infiltration, and promoted repair of damaged nerve fibers. Overall, these findings suggest that GeXIVA[1,2] exerts analgesic effects through anti-inflammatory and neuroprotective mechanisms, providing a potential new therapeutic strategy for diabetic neuropathic pain and postherpetic neuralgia.

## 1. Introduction

Chronic neuropathic pain, caused by damage or disease in the peripheral or central nervous system, is a persistent condition that typically lasts for more than three months and is characterized by tingling and burning sensations, often leading to paroxysmal or spontaneous pain [1,2]. Among various forms of chronic neuropathic pain, diabetic neuropathic pain and postherpetic neuralgia both result from the progression of underlying diseases. Diabetic neuropathic pain arises from nerve damage due to metabolic disturbances caused by long-term hyperglycemia [3], while postherpetic neuralgia results from the reactivation of latent varicella–zoster virus in the dorsal root (spinal) ganglia, followed by viral spread along sensory nerves that can also affect peripheral sensory nerve terminals, leading to persistent neuroinflammatory responses and nerve injury [4]. Hyperglycemia and viral invasion damage peripheral nerve tissues, leading to structural neuronal impairments, including abnormal curling and deformation of the myelin sheath, formation of scar tissue, and other pathological changes, ultimately resulting in peripheral nerve dysfunction. Additionally, immune imbalances around the affected nerves induce sustained inflammatory reactions, accompanied by the excessive release of inflammatory cytokines, which further exacerbate nerve damage and intensify pain. Diabetic neuropathic pain and postherpetic neuralgia exhibit high incidence rates (affecting approximately 50% of diabetic patients [5] and 10–20% of herpes zoster patients [6]), and typically present with mechanical hyperalgesia [7,8]. The associated pain can lead to severe sleep disturbances, depressed mood, and diminished quality of life for patients [9]. Compared to neuropathic pain resulting from trauma or surgery, diabetic neuropathic pain and postherpetic neuralgia are more intractable, involving non-single-event nerve damage and are characterized by chronicity and recurrent episodes. Therefore, diabetic neuropathic pain and postherpetic neuralgia remain persistent challenges in pain management.

Current clinical pharmacotherapies for diabetic neuropathic pain and postherpetic neuralgia include tricyclic antidepressants (e.g., amitriptyline, duloxetine) [10], anticonvulsants (e.g., carbamazepine) [11], antiepileptic drugs (e.g., gabapentin, pregabalin), and opioids (e.g., morphine sulfate, oxycodone). These agents primarily function by reducing the release of excitatory neurotransmitters and inhibiting pain signal transmission. However, their efficacy is limited, and they are associated with side effects such as dizziness, vomiting, drowsiness, and urinary retention. In severe cases, they may even cause respiratory and central nervous system depression, exhibiting dose-limiting toxicity [12]. Consequently, there is a pressing need to develop novel therapeutics with improved safety profiles and superior analgesic efficacy for diabetic neuropathic pain and postherpetic neuralgia treatment.

In recent years, numerous novel analgesic strategies have emerged to overcome the limitations of conventional drugs, including botulinum toxin type A (BTX-A) [13], vixotrigine (a sodium channel blocker) [14], and eliapixant (a selective P2X3 receptor antagonist) [15]. Among these, conotoxins, derived from marine cone snails, are promising candidates for treating chronic neuropathic pain due to their high selectivity for ion channels involved in pain signaling [16]. ω-conotoxin MVIIA (ziconotide, Prialt) has received FDA approval for managing severe chronic neuropathic pain [17], serving as a successful example of conotoxin-based drug development. However, as ziconotide’s target (N-type calcium channels) is located in the central nervous system, it requires intrathecal administration, which poses certain limitations [18]. Thus, there remains a need to develop novel conotoxins that are safer, more selective, and amenable to simpler administration.

GeXIVA is a novel αO-conotoxin identified from cone snails (Generalis) in the South China Sea, which selectively blocks the α9α10 nicotinic acetylcholine receptor (α9α10 nAChR). α9α10 nAChR is associated with the peripheral nervous and immune systems, thus intramuscular administration can yield excellent analgesic effects, obviating the need for intrathecal injection. Our research group successfully synthesized GeXIVA[1,2] [19] and demonstrated its potent analgesic efficacy in chemotherapy-induced peripheral neuropathy (CIPN) [20,21], spared nerve injury (SNI), and chronic constriction injury (CCI) [22,23] without inducing tolerance or addiction. Furthermore, studies on its biosafety, pharmacokinetic profile, and structural stability [24,25,26] indicate that GeXIVA[1,2] possesses a favorable safety profile, maintains analgesic effects for up to 6 h, and exhibits good stability under normal conditions. Therefore, GeXIVA[1,2] holds significant promise for treating neuropathic pain.

Due to diabetic neuropathic pain and postherpetic neuralgia involving complex pathogenesis distinct from reported chronic neuropathic pain (i.e., CIPN, SNI and CCI), they require therapeutic intervention of extended duration. More importantly, existing literature has not explored the potential restorative effects of GeXIVA on peripheral neuropathy. Therefore, investigating the therapeutic efficacy of GeXIVA[1,2] in diabetic neuropathic pain and postherpetic neuralgia, along with the associated physiological and pathological changes post-treatment, is of great significance. This study aims to elucidate the therapeutic mechanisms of GeXIVA[1,2] against diabetic neuropathic pain and postherpetic neuralgia by exploring its dose–response relationship, therapeutic effects, and its role in repairing neuropathological structures, modulating immune cell population balance, and regulating cytokine release pathways. Additionally, in reported studies, GeXIVA[1,2] has been administered via intramuscular injection, which can cause tissue damage and infection, thereby hindering self-administration [27]. This study will investigate the therapeutic efficacy of subcutaneously administered GeXIVA[1,2]. Subcutaneous injection is a simpler procedure that patients can perform independently, which can improve compliance [28] and is also conducive to the development of new drug formulations, such as microneedles and hypodermic implants [23].

In this study, we systematically evaluated the analgesic and neurorestorative effects of GeXIVA[1,2] in diabetic neuropathic pain and postherpetic neuralgia. We first determined the dosage and analgesic duration via a single administration. Subsequently, once-daily subcutaneous injections were administered to observe the analgesic effects and its improvement of motor function, based on mechanical pain threshold and behavioral performance. Building on this, we further examined changes in inflammatory cytokine levels, immune cell infiltration, and neural structure in the model animals following GeXIVA[1,2] treatment to investigate the associated physiological and pathological alterations.

## 2. Results

### 2.1. Dose–Response Relationship

**Figure 1:**
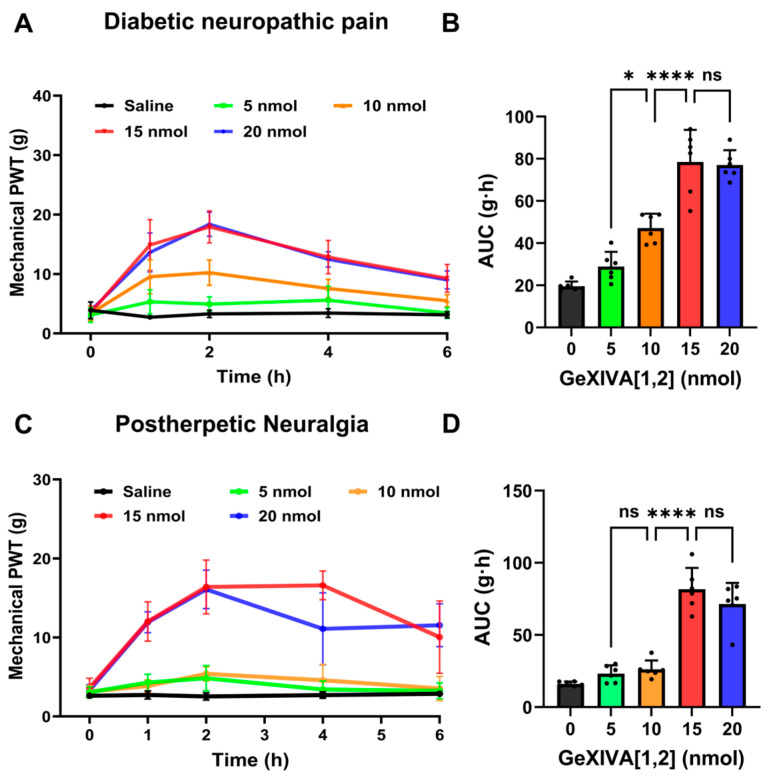
Dose–response relationship of the analgesic effects of GeXIVA[1,2] in diabetic neuropathic pain and postherpetic neuralgia rats. (**A**) Time-effect relationship and dose–response relationship of the analgesic effect following a single subcutaneous injection of GeXIVA[1,2] in diabetic neuropathic pain rats. (**B**) Area under the curve (AUC) of the corresponding dose within 6 h after injection, calculated from the curves in Figure 1A. (**C**) Time-effect relationship and dose–response relationship of the analgesic effect following a single subcutaneous injection of GeXIVA[1,2] in postherpetic neuralgia rats. (**D**) Area under the curve (AUC) of the corresponding dose within 6 h after injection, calculated from the curves in Figure 1C. * indicates *p* < 0.05, **** indicates *p* < 0.0001, ns indicates no significant difference; *n* = 6.

To investigate the dose–response relationship of GeXIVA[1,2] in treating diabetic neuropathic pain and postherpetic neuralgia, we selected rats with normal mechanical pain thresholds to establish diabetic neuropathic pain and postherpetic neuralgia models. The diabetic neuropathic pain model was induced by intraperitoneal injection of streptozocin (STZ) solution (40 mg/kg) for three days, while the postherpetic neuralgia model was established by a single intraperitoneal injection of resiniferatoxin (RTX) solution (200 μg/kg). A significant decrease in the paw withdrawal threshold (PWT) indicated successful model induction. Following modeling, changes in the mechanical PWT of diabetic neuropathic pain rats at 1, 2, 4, and 6 h after subcutaneous administration of GeXIVA[1,2] were measured using von Frey filaments. As shown in Figure 1A, the analgesic effect of GeXIVA[1,2] emerged gradually over time, with the PWT showing an upward trend and peaking at 2 h post-injection. Compared to the saline group, the 5 nmol and 10 nmol groups exhibited relatively weak analgesic effects, *p* (5 nmol) = 0.2086, *p* (10 nmol) = 0.0029. In contrast, the 15 nmol and 20 nmol groups demonstrated strong analgesic effects at 2 h after administration, *p* (15 nmol) = 0.0002, *p* (20 nmol) < 0.0001), and the analgesic activity persisted for up to 4 h (*p* < 0.05).

The area under the curve (AUC) of PWT from 0 to 6 h was calculated to analyze the dose–response relationship (Figure 1B). The results indicated that, within the range of 0–15 nmol, higher doses produced more significant analgesic effects. The 15 nmol group was significantly different from the 10 nmol group (*p* < 0.05), while no significant difference was observed between the 15 nmol and 20 nmol groups.

The changes in PWT of postherpetic neuralgia rats before and after drug administration were assessed in the same manner. As shown in Figure 1C, PWT in all GeXIVA[1,2]-treated groups showed an increasing trend compared to saline group, indicating its analgesic effect. The 5 nmol group exhibited weak analgesic effects, peaking of PWT at 2 h, but showed no significant difference compared to the saline group *p* (5 nmol) = 0.1604. The 15 nmol and 20 nmol groups significantly increased PWT as early as 1 h post-injection and demonstrated strong analgesic effects at 2 h, with activity lasting up to 6 h. The AUC of the 0–6 h showed that within the range of 0–15 nmol, a higher dose led to a more significant analgesic effect (Figure 1D). The 15 nmol group showed a significant different compared to the saline group (*p* < 0.0001) and was significantly different from the 10 nmol group (*p* < 0.0001), but no significant difference was found compared to the 20 nmol group (*p* = 0.9062).

The results of the dose–response relationship testing indicate that GeXIVA[1,2] exerts analgesic effects in both diabetic neuropathic pain and postherpetic neuralgia rat, and its efficacy is closely related to dose and time. 15–20 nmol GeXIVA[1,2] per rat produced significant analgesic effects. Overall, the 15 nmol dose is the optimal choice, and the PWT measurement should be conducted at 2 h post-injection. However, this experiment only evaluated the effect of a single subcutaneous injection, and its analgesic effect of sustained treatment remains to be further investigated.

### 2.2. The Analgesic Effect of Sustained Treatment with GeXIVA[1,2]

**Figure 2:**
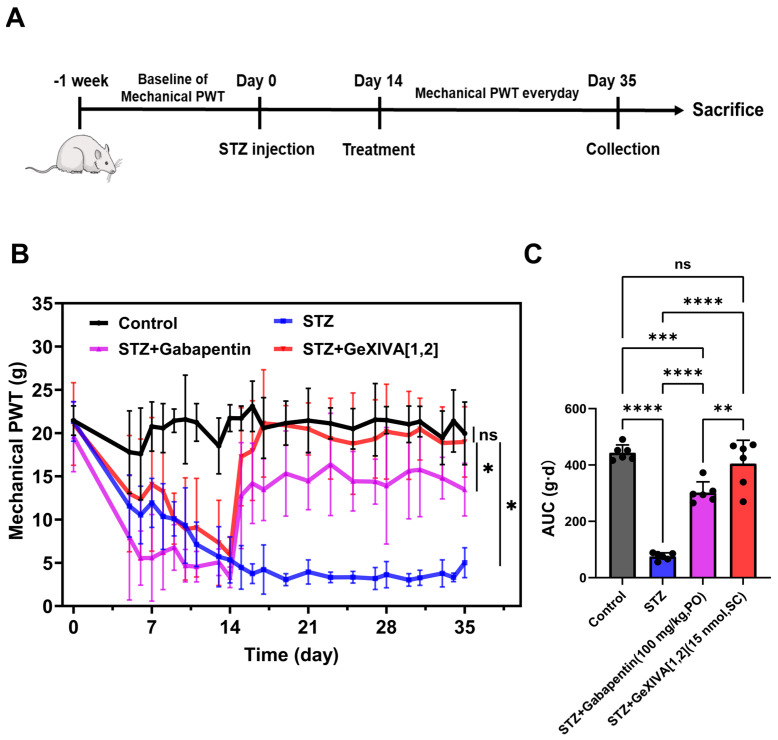
The analgesic effect of sustained treatment with GeXIVA[1,2] in diabetic neuropathic pain rats. (**A**) Experimental timeline. (**B**) Effect of GeXIVA[1,2] on the mechanical pain threshold in diabetic neuropathic pain rats (treatment initiated from Day 14). (**C**) AUC for different groups. * indicates *p* < 0.05, ** indicates *p* < 0.01, *** indicates *p* < 0.001, **** indicates *p* < 0.0001, ns indicates no significant difference; *n* = 6.

**Figure 3:**
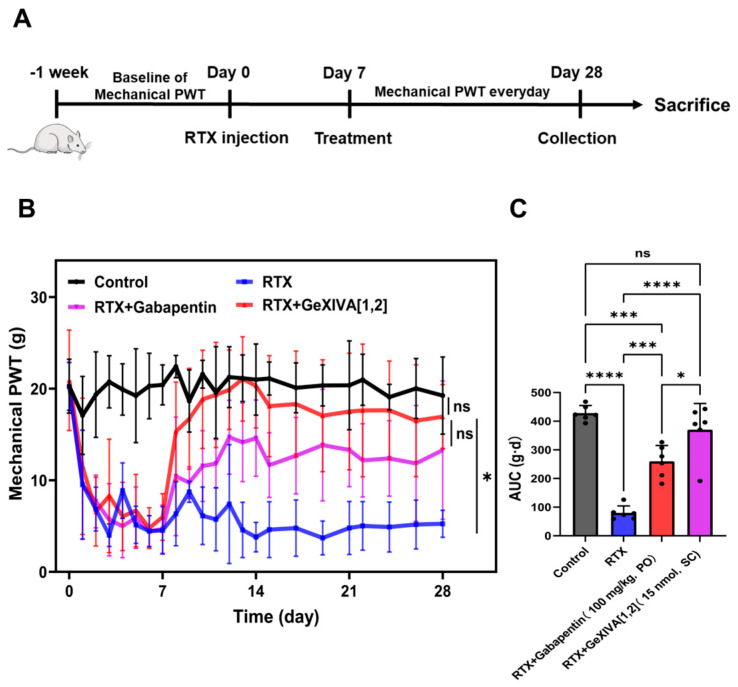
The analgesic effect of sustained treatment with GeXIVA[1,2] in postherpetic neuralgia rats. (**A**) Experimental timeline. (**B**) Effect of GeXIVA[1,2] on the mechanical pain threshold in postherpetic neuralgia rats (treatment initiated from Day 7). (**C**) AUC for different groups. * indicates *p* < 0.05, *** indicates *p* < 0.001, **** indicates *p* < 0.0001, ns indicates no significant difference; *n* = 6.

In the dose–response study, we established the efficacy of a single subcutaneous injection of GeXIVA[1,2]. We monitored changes in mechanical pain sensitivity in diabetic neuropathic pain and postherpetic neuralgia model rats before and after treatment via the PWT test (Figure 2A and Figure 3A). Rats were divided into four groups: a control group (healthy rats), an STZ group (diabetic neuropathic pain model rats without treatment), an STZ + Gabapentin group (diabetic neuropathic pain model rats treated with the clinically used positive-control drug Gabapentin [29,30,31]), and an STZ + GeXIVA[1,2] group (diabetic neuropathic pain model rats treated with GeXIVA[1,2]). Baseline PWT was measured after grouping. Rats in the diabetic neuropathic pain model groups received intraperitoneal injections of STZ (40 mg/kg) for three consecutive days. After 72 h, blood glucose levels were measured. Blood glucose increased from 6.2 ± 0.72 mmol/L to 20 ± 8.8 mmol/L (Figure S1), and PWT decreased from 21 ± 2.3 g (Day 0) to 5.3 ± 2.7 g (Day 14, *p* < 0.05 compared to the control group), indicating successful establishment of the diabetic neuropathic pain model. Treatment began in the STZ + Gabapentin and STZ + GeXIVA[1,2] groups 14 days after STZ injection. On the first day of treatment, both treatment groups showed significant pain relief. PWT increased from 4.3 ± 1.2 g to 12 ± 6.1 g in the STZ + Gabapentin group, and from 5.9 ± 2.0 g to 17 ± 5.7 g in the STZ + GeXIVA[1,2] group. After three weeks (21 days) of once-daily treatment, the PWT in the GeXIVA[1,2]-treated group recovered to 18 ± 4.0 g. This analgesic effect was significantly superior to that in the Gabapentin-treated group (13 ± 3.0 g) (*p* < 0.05) and showed no statistically significant difference compared to the control group (19 ± 3.6 g) (*p* = 0.6653). These results indicate that both GeXIVA[1,2] and Gabapentin possess analgesic effects, with GeXIVA[1,2] demonstrating a more potent effect than the clinically used analgesic drug Gabapentin. Following GeXIVA[1,2] treatment, the pain threshold in rats recovered to a level comparable to that of healthy rats (Figure 2B). To quantify the degree of analgesic effects, the AUC was calculated as a cumulative measure of the pain threshold. The results showed that the AUC after GeXIVA[1,2] treatment was significantly higher than that after Gabapentin treatment (*p* < 0.05), further confirming the significant analgesic efficacy of GeXIVA[1,2] (Figure 2C). However, the three diabetic neuropathic pain model groups exhibited a mild decrease in body weight (Figure S2A). Treatment with either GeXIVA[1,2] or gabapentin resulted in no significant difference in body weight compared to the untreated group (*p* > 0.05), indicating that the treatment primarily alleviates neuropathic symptoms rather than intervening in the pathological progression of diabetes itself.

To evaluate the sustained therapeutic effect of GeXIVA[1,2] on postherpetic neuralgia, all rats in postherpetic neuralgia model groups developed significant peripheral neuropathic pain one week after RTX injection, as evidenced by a marked decrease in the PWT. The PWT in the RTX group decreased from 20 ± 1.8 g (Day 0) to 6.0 ± 1.8 g (Day 7, *p* < 0.05 vs. control). Similarly, significant reductions were observed in the RTX + Gabapentin group (from 20 ± 2.6 g to 4.7 ± 2.6 g, *p* < 0.05 vs. control) and the RTX + GeXIVA[1,2] group (from 21 ± 5.5 g to 6.0 ± 2.6 g, *p* < 0.05 vs. control), confirming the successful establishment of the postherpetic neuralgia model. On the first day of treatment (Day 7), the RTX + GeXIVA[1,2] group exhibited significant pain relief, with the PWT increasing to 15 ± 5.4 g. Following 21 days of consecutive daily administration, the PWT in the GeXIVA[1,2]-treated group recovered to 17 ± 3.5 g, showing no statistically significant difference compared to the control group (*p* > 0.05). This indicates that the pain threshold in rats treated with GeXIVA[1,2] had returned to a level comparable to that of healthy rats (Figure 3B). To quantify the extent of pain relief, we calculated the AUC for different groups as a cumulative measure of the PWT. The AUC for the RTX + GeXIVA[1,2] group was significantly higher than that for both the RTX group and the RTX + Gabapentin group. This result demonstrates that GeXIVA[1,2] significantly alleviated pain in postherpetic neuralgia rats, and its analgesic effect was superior to that of clinically used Gabapentin (Figure 3C). Furthermore, GeXIVA[1,2] did not induce body weight loss in postherpetic neuralgia rats, indicating a favorable safety feature (Figure S2B).

In summary, GeXIVA[1,2] demonstrated a stronger analgesic effect against diabetic neuropathic pain and postherpetic neuralgia than Gabapentin, characterized by rapid onset and sustained, stable analgesic properties. Its efficacy remained stable after 21 days of continuous administration without signs of analgesic tolerance, highlighting its good safety profile and potential for long–term use. These findings suggest that GeXIVA[1,2] holds significant promise for the sustained treatment of chronic neuropathic pain.

### 2.3. Effect of GeXIVA[1,2] on Motor Dysfunction Induced by Neuropathic Pain

**Figure 4:**
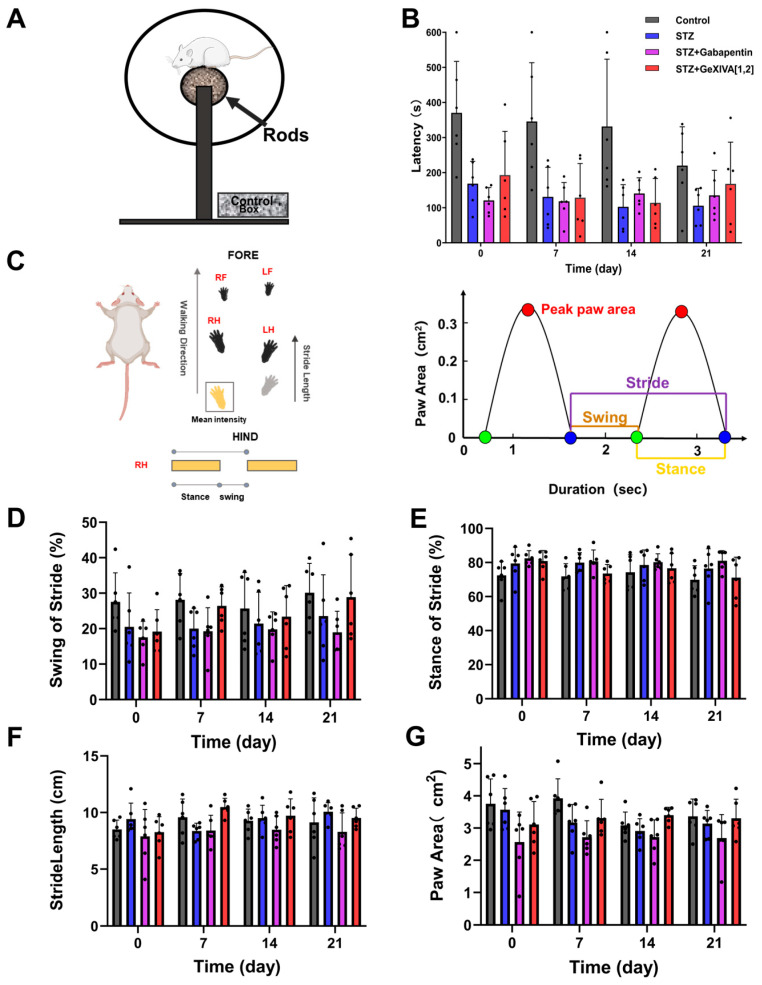
Effect of GeXIVA[1,2] on motor dysfunction induced by diabetic neuropathic pain. (**A**) Schematic diagram of the rotarod test. (**B**) Latency to fall from the rotating rod, *n* = 6. (**C**) Schematic diagram of the gait test and representation of gait parameters. RH = right hind limb, LH = left hind limb. (**D**–**G**) Gait changes in rats after 1, 2, and 3 weeks of sustained treatment, *n* = 6.

Peripheral neuropathy impairs the normal function of both sensory and motor nerves, leading to motor deficits and reduced coordination [32]. We employed the Rotarod test (Figure 4A) to assess motor coordination in diabetic neuropathic pain rats and to investigate whether GeXIVA[1,2] has a therapeutic effect on motor function (Figure 4B). Prior to treatment (post-modeling, Day 0), the latency to fall in the STZ group (170 ± 58 s) was shorter than that in normal rats (350 ± 145 s), indicating impaired motor coordination in diabetic neuropathic pain rats. During the treatment period (Days 7, 14, and 21), no increase in latency was observed in the treated groups. After 21 days of treatment, the latency was 104 ± 45 s in the STZ group and 149 ± 119 s in the STZ + GeXIVA[1,2] group. However, no statistically significant difference was found between the treated group and the diabetic neuropathic pain model group, suggesting that GeXIVA[1,2] may not significantly improve motor coordination in diabetic neuropathic pain rats. Peripheral neuropathy often induces abnormal gait alterations [33]. We used a gait analysis system to quantitatively analyze changes in locomotor patterns. Parameters including swing time, stance time, stride length, and paw print area were recorded (Figure 4C). The results showed that diabetic neuropathic pain rats had shorter swing times and longer stance times compared to normal rats. But no significant differences were observed following GeXIVA[1,2] treatment. Furthermore, neither stride length nor paw print area showed significant changes after treatment, indicating that GeXIVA[1,2] did not significantly affect gait parameters in diabetic neuropathic pain rats, a result consistent with Gabapentin treatment (Figure 4D–G).

**Figure 5:**
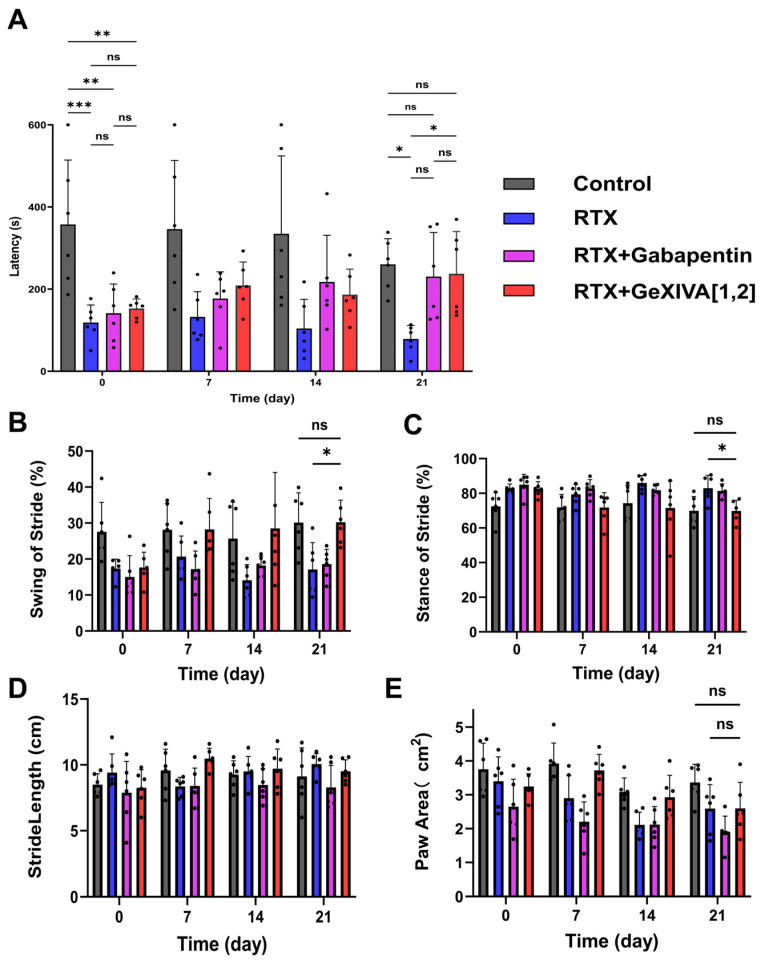
Effect of GeXIVA[1,2] on motor dysfunction induced by postherpetic neuralgia. (**A**) Latency to fall from the rotating rod. (**B**–**E**) Gait changes in rats after 1, 2, and 3 weeks of sustained treatment. * indicates *p* < 0.05, ** indicates *p* < 0.01, *** indicates *p* < 0.001, ns indicates no significant difference; *n* = 6.

We also investigated the effects of GeXIVA[1,2] on motor coordination and gait parameters in postherpetic neuralgia rats. As shown in Figure 5A, prior to treatment (Day 0), all postherpetic neuralgia model groups (RTX, RTX + Gabapentin, RTX + GeXIVA[1,2]) exhibited significantly shorter latencies to fall compared to normal rats (*p* < 0.05), indicating reduced motor coordination in postherpetic neuralgia rats. After 21 days of treatment, the latency increased from 141 ± 71 s to 230 ± 108 s in the RTX + Gabapentin group, and from 153 ± 23 s to 211 ± 100 s in the RTX + GeXIVA[1,2] group. In contrast, the latency in the untreated RTX group decreased from 118.4 ± 43.08 s to 71 ± 38 s. At the end of the treatment period (Day 21), postherpetic neuralgia rats treated with GeXIVA[1,2] showed a significant difference compared to untreated postherpetic neuralgia rats, while no significant difference was observed compared to the control or Gabapentin-treated groups. This result suggests that GeXIVA[1,2] treatment can improve motor coordination in postherpetic neuralgia rats, restoring the latency to a level comparable to normal. Gait parameters in postherpetic neuralgia rats were also tested. As shown in Figure 5B, after treatment, the swing time percentage in the RTX + GeXIVA[1,2] group increased from 18 ± 4.3% to 30 ± 6.1%, showing a significant difference compared to the RTX group (Day 21: 17 ± 7.5%, *p* < 0.05). Correspondingly, the stance time percentage in the RTX + GeXIVA[1,2] group showed a decreasing trend (from 82 ± 4.3% to 70 ± 6.2%, Figure 5C). However, no significant differences were found in stride length or paw print area (*p* > 0.05, Figure 5D,E). The observed changes in stance and swing times may be attributed to enhanced voluntary movement due to pain relief following treatment. Previous studies have evaluated GeXIVA[1,2] administered alone in the rotarod test and demonstrated that it does not impair normal locomotor activity in rats [22]. Consistent with these findings, our results showed that GeXIVA[1,2] did not adversely affect motor coordination in either diabetic neuropathic pain or postherpetic neuralgia models. Moreover, GeXIVA[1,2] improved motor coordination in postherpetic neuralgia rats, restoring performance toward normal levels, while exerting minimal effects on specific gait parameters such as stride length and paw print area.

### 2.4. Analysis of Inflammatory Cytokines in Injured Nerves and Perineural Tissues

**Figure 6:**
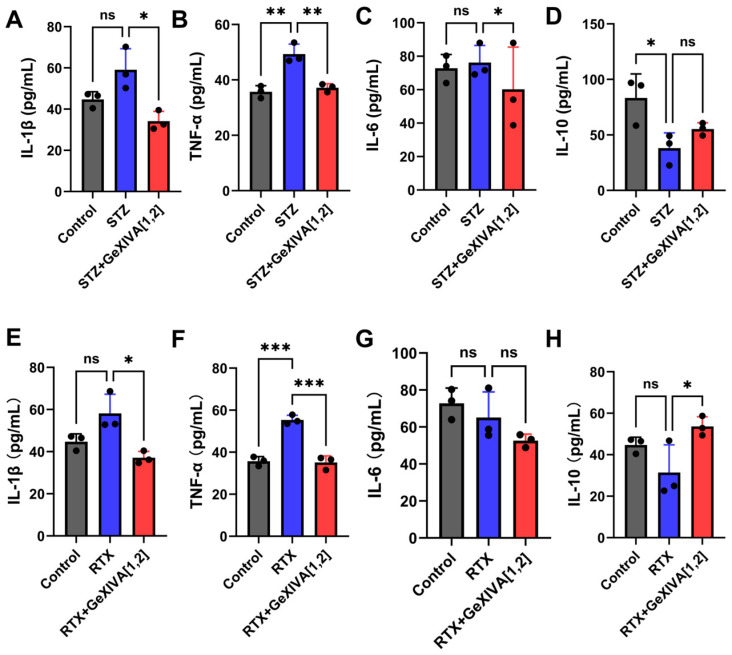
Inflammatory cytokines in injured nerves and perineural tissues. (**A**–**D**) Effects of GeXIVA[1,2] treatment on the levels of IL-1β, TNF-α, IL-6, and IL-10 in tissue homogenates of diabetic neuropathic pain rats. (**E**–**H**) Effects of GeXIVA[1,2] treatment on the levels of IL-1β, TNF-α, IL-6, and IL-10 in tissue homogenates of postherpetic neuralgia rats. * indicates *p* < 0.05, ** indicates *p* < 0.01, *** indicates *p* < 0.001, ns indicates no significant difference.

Inflammatory cytokines play a key role in the pathological mechanism of neuropathic pain. To evaluate the role of cytokines in pain sensitization, in animal models of diabetic neuropathic pain and postherpetic neuralgia we measured the levels of key inflammatory factors (i.e., IL-1β, TNF-α, IL-6, and IL-10) in injured nerves and surrounding tissues using ELISA (Figure S3). At the same time, we investigated the regulatory effects of GeXIVA[1,2] on neuroinflammation. After 21 days of treatment, Figure 6A–C shows that GeXIVA[1,2] treatment downregulated the pro-inflammatory cytokines IL-1β, TNF-α, and IL-6: IL-1β decreased from 0.25 ± 0.03 pg/mL to 0.13 ± 0.03 pg/mL, TNF-α decreased from 0.26 ± 0.03 pg/mL to 0.17 ± 0.01 pg/mL, and IL-6 decreased from 0.30 ± 0.04 pg/mL to 0.18 ± 0.03 pg/mL. These changes were significantly different from the STZ group (*p* < 0.05), with IL-1β and TNF-α restored to levels comparable to those in healthy rats. IL-10, an anti-inflammatory cytokine that can suppress neuroinflammation and reduce neuronal damage, did not show a significant increase after treatment (Figure 6D). In postherpetic neuralgia model rats, we similarly observed that the RTX + GeXIVA[1,2] group exhibited significantly lower levels of IL-1β and TNF-α compared to the RTX group (Figure 6E,F). IL-1β decreased from 0.28 ± 0.06 pg/mL to 0.15 ± 0.02 pg/mL, and TNF-α decreased from 0.31 ± 0.02 pg/mL to 0.15 ± 0.02 pg/mL, both returning to levels seen in healthy rats. IL-6 also showed a downward trend (Figure 6G), decreasing from 0.26 ± 0.06 pg/mL to 0.21 ± 0.01 pg/mL. However, IL-10 only slightly increased in the treatment group (Figure 6H), without reaching statistical significance (*p* > 0.05). In both neuropathic pain models, GeXIVA[1,2] demonstrated an inhibitory effect on the release of pro-inflammatory cytokines, while its impact on the anti-inflammatory cytokine IL-10 was not significant.

### 2.5. Infiltration of Immune Cells Around Injured Nerves

**Figure 7:**
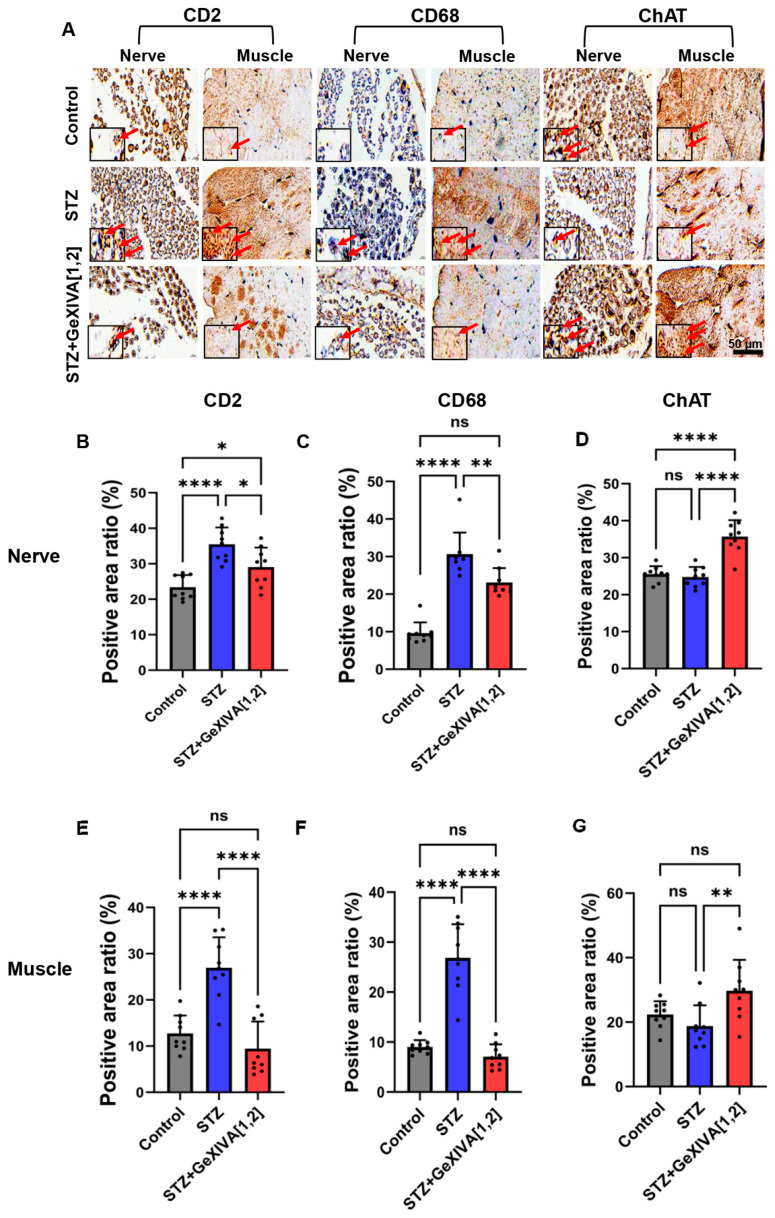
Infiltration of immune cells around the injured nerve in diabetic neuropathic pain rats. (**A**) Representative immunohistochemical staining images of the sciatic nerve and surrounding muscle tissue using antibodies against CD2, CD68, and ChAT. Brown staining indicates positive cells (red arrows), and blue staining marks the nuclei. (**B**–**D**) Percentages of CD2-, CD68-, and ChAT-positive cells in the sciatic nerve. (**E**–**G**) Percentages of CD2-, CD68-, and ChAT-positive cells in the surrounding muscle tissue. * indicates *p* < 0.05, ** indicates *p* < 0.01, **** indicates *p* < 0.0001, ns indicates no significant difference.

**Figure 8:**
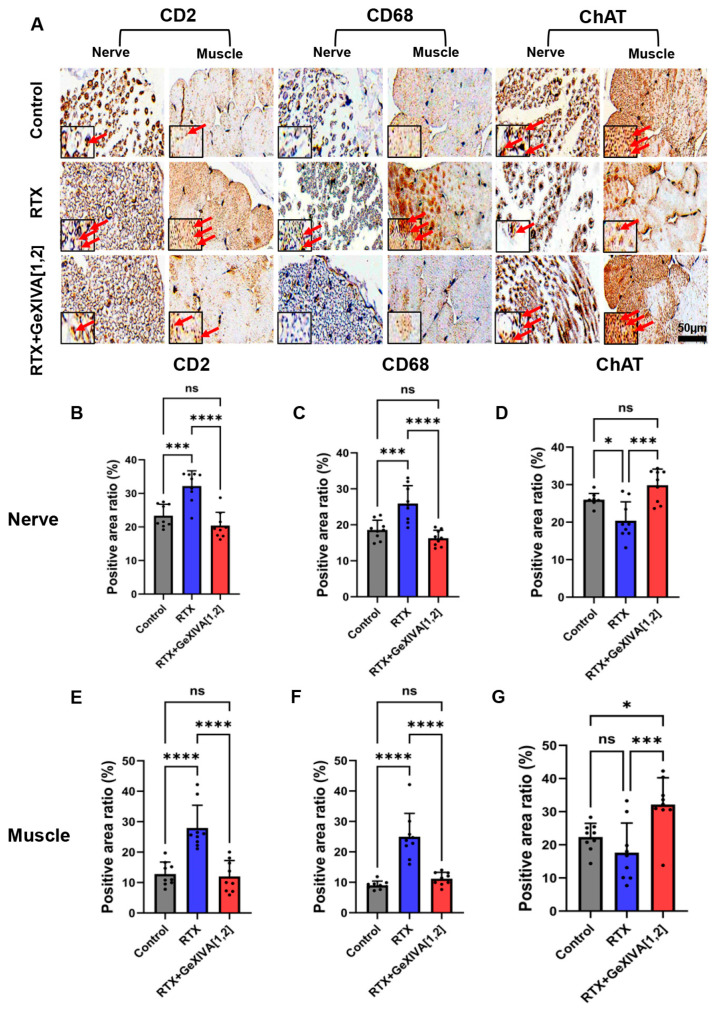
Infiltration of immune cells around the injured nerve in postherpetic neuralgia rats. (**A**) Representative immunohistochemical staining images of the sciatic nerve and surrounding muscle tissue using antibodies against CD2, CD68, and ChAT. Brown staining indicates positive cells (red arrows), and blue staining marks the nuclei. (**B**–**D**) Percentages of CD2-, CD68-, and ChAT-positive cells in the sciatic nerve. (**E**–**G**) Percentages of CD2-, CD68-, and ChAT-positive cells in the surrounding muscle tissue. * indicates *p* < 0.05, *** indicates *p* < 0.001, **** indicates *p* < 0.0001, ns indicates no significant difference.

Neuropathic pain can arise from metabolic injury or physical damage to the peripheral sensory system; these conditions drive pathological changes in immune cells and neurons at the site of injury [34]. Excessive immune cells release a large number of inflammatory mediators, further exacerbating neuroinflammation and nerve damage. Meanwhile, injury or loss of function in cholinergic neurons can impair nerve conduction efficiency. Therefore, we assessed immune cell infiltration and function in cholinergic neurons in the peripheral nerves and the muscle tissue surrounding the injured nerves of diabetic neuropathic pain and postherpetic neuralgia rats by examining the expression of CD2, CD68, and ChAT via immunohistochemical staining. In diabetic neuropathic pain rats, significant immune cell infiltration and neuronal damage were observed in the sciatic nerve and adjacent muscle tissue (Figure 7A). Quantitative analysis revealed that in the sciatic nerve, the levels of CD2 and CD68 were significantly higher in the STZ group compared to the control group (Figure 7B,C), indicating substantial macrophage infiltration, while ChAT expression showed no significant change (Figure 7D). Compared to the STZ group, treatment with GeXIVA[1,2] significantly reduced the levels of both CD2 and CD68 in the sciatic nerve and increased ChAT expression. Abnormal immune cell infiltration was also observed in the adjacent muscle tissue. The levels of CD2, CD68, and ChAT were significantly higher in the STZ group than in the control group (Figure 7E–G), while the GeXIVA[1,2]-treated rats showed no significant difference from the control group. In postherpetic neuralgia rats, differences in immunohistochemical staining among the groups were visually apparent (Figure 8A). Untreated postherpetic neuralgia rats exhibited significant immune cell infiltration (elevated CD2 and CD68) in both the sciatic nerve and adjacent muscle, which showed no statistical difference from normal rats after treatment (Figure 8B,C,E,F). Concurrently, the restorative effect of treatment on ChAT expression was relatively limited in nerve tissue (Figure 8D) but more pronounced in muscle tissue (Figure 8G). These results demonstrate that GeXIVA[1,2] can inhibit the infiltration of immune cells around injured nerves.

### 2.6. Pathological Structural Analysis of Sciatic Nerve

**Figure 9:**
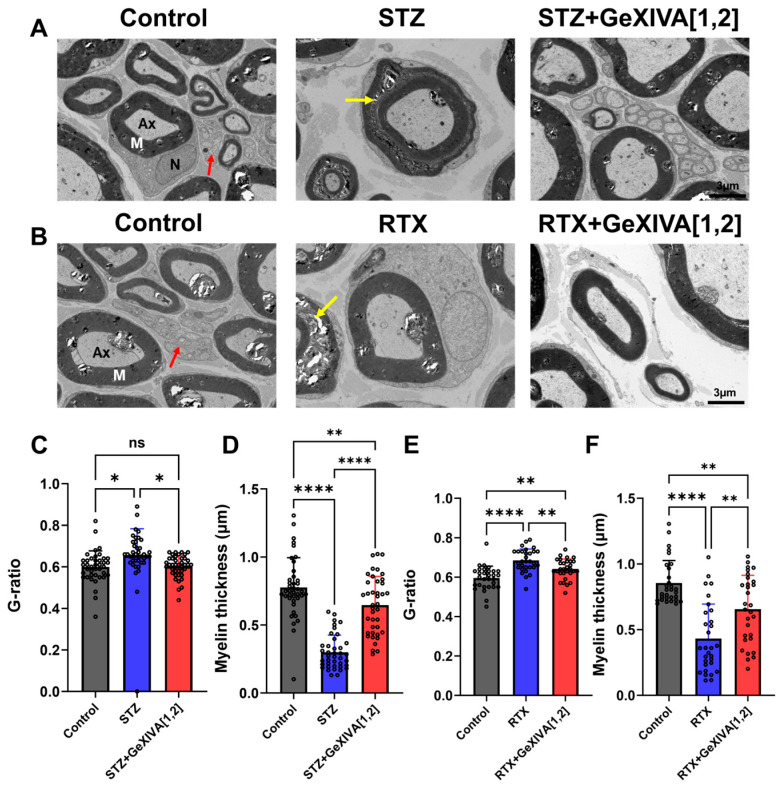
Ultrastructural and quantitative analysis of sciatic nerves in diabetic neuropathic pain and postherpetic neuralgia rats. (**A**) Images of ultrastructure of the sciatic nerve in diabetic neuropathic pain rats under transmission electron microscopy (TEM). (**B**) Images of ultrastructure of the sciatic nerve in postherpetic neuralgia rats under TEM. Ax, axon; M, myelin sheath; N, Schwann cell nucleus. Yellow arrows indicate lamellar separation between the axon and myelin sheath and demyelination; red arrows indicate unmyelinated neurons. (**C**) Quantitative analysis of the G-ratio (axon diameter/nerve fiber diameter) in sciatic nerves of diabetic neuropathic pain rats. An increased G-ratio reflects myelin thinning or loss. (**D**) Quantitative analysis of myelin sheath thickness in sciatic nerves of diabetic neuropathic pain rats. Thicker myelin is generally associated with faster nerve conduction velocity. (**E**,**F**) Quantitative analysis of the G-ratio and myelin sheath thickness in sciatic nerves of postherpetic neuralgia rats. * indicates *p* < 0.05, ** indicates *p* < 0.01, **** indicates *p* < 0.0001, ns indicates no significant difference.

The myelin sheath facilitates efficient nerve impulse propagation and provides axonal protection. Peripheral nerve injury can lead to demyelination, fragmentation and axonal retraction [35]. In diabetic neuropathic pain, chronic hyperglycemia increases oxidative stress, damaging Schwann cells and resulting in myelin fragmentation and focal loss [36]. In the RTX-induced postherpetic neuralgia rat model, myelinated fibers in the sciatic nerve are significantly impaired, exhibiting axonal swelling, myelin loosening, and disruption [37]. Nerve fibers rely on saltatory conduction, and changes in axon diameter can affect conduction velocity and action potential firing [38]. Abnormal action potential discharge, in turn, can induce hyperalgesia [39]. Transmission Electron Microscopy (TEM) allows direct visualization of ultrastructural changes in the sciatic nerve. Quantitative analysis of the G-ratio and myelin thickness was performed to assess the structural integrity and conductive functional state of nerve fibers, thereby investigating the potential neuroprotective effects of GeXIVA[1,2]. We first evaluated myelin changes in the sciatic nerves of diabetic neuropathic pain rats (Figure 9A). TEM images revealed intact nerve fiber structures with regularly arranged axons and myelin in control group. In contrast, the STZ group showed evident myelin degradation, structural collapse, and demyelination (indicated by yellow arrows in Figure 9A, STZ panel). Comparatively, GeXIVA[1,2] treatment resulted in better-preserved myelin morphology and improved myelin thickness. Quantitative analysis demonstrated that the STZ group had a significantly higher G-ratio and reduced myelin thickness compared to the control group. The STZ + GeXIVA[1,2] group showed no significant difference in G-ratio from the control, and myelin thickness was markedly increased compared with STZ group, approaching control levels (Figure 9C,D). Similarly, we examined the sciatic nerve ultrastructure in postherpetic neuralgia rats. The RTX group exhibited ultrastructural damage to the sciatic nerve, while the RTX + GeXIVA[1,2] group displayed better myelin morphology (Figure 9B). Quantitative results indicated that GeXIVA[1,2] treatment significantly changes G-ratio and myelin thickness compared to the RTX group, although values did not fully return to normal levels (Figure 9E,F). These findings indicate that GeXIVA[1,2] treatment promotes the restoration of myelin sheath structure. GeXIVA[1,2] treated nerves showed no obvious lamellar separation or myelin collapse, with more orderly arrangement, a decreased G-ratio, and increased myelin thickness.

## 3. Discussion

Previous studies have demonstrated that GeXIVA[1,2] can alleviate oxaliplatin-induced chemotherapy-related neuropathic pain [21] and trauma-induced neuropathic pain [23]. In this study, we investigated the therapeutic efficacy of GeXIVA[1,2] against disease-induced diabetic neuropathic pain and postherpetic neuralgia, and explored the pathological changes in nerve tissues following treatment. We assessed the impact of GeXIVA[1,2] on motor function through animal behavioral tests. The rotarod test primarily reflects motor coordination. In our study, diabetic neuropathic pain rats showed a decreased latency to fall from the rotarod, consistent with reported research [40]. However, GeXIVA[1,2] treatment did not restore this latency to the level of healthy rats, which may be related to the dosage used [41]. Furthermore, reported studies indicated that diabetic neuropathic pain can lead to gait abnormalities and increase fall risk [33].

Existing studies on the gait of diabetic neuropathic pain rats observed that diabetic neuropathic pain rats exhibited reduced paw contact area and decreased stride length [42], while pharmacological treatment for diabetic neuropathic pain alleviated pain but did not improve gait dysfunction [43]. Consequently, we employed a gait analysis system to quantitatively analyze changes in the locomotor patterns of diabetic neuropathic pain rats, revealing no significant changes before and after GeXIVA[1,2] treatment. This suggests that analgesic effects and motor function improvement are not entirely concordant, and drugs may not directly correct gait abnormalities when alleviating pain. In the postherpetic neuralgia model, GeXIVA[1,2] treated postherpetic neuralgia rats showed a significantly increased latency to fall on the rotarod, recovering to a level comparable to healthy rats, indicating that GeXIVA[1,2] improves balance and motor coordination. However, its effect on improving gait in postherpetic neuralgia rats was not pronounced. Currently, no systematic studies have reported changes in gait parameters or the effects of drug intervention in postherpetic neuralgia animal models. Therefore, our experimental results may provide new insights for gait research in postherpetic neuralgia models. The gait changes may depend on the type of neuropathic pain conditions. Studies on chronic constriction injury and spared nerve injury models have shown significant gait parameter abnormalities in model animals, which can be partially alleviated by drug treatment [44,45]. In contrast, the lack of marked gait parameter changes in our study might be related to the absence of direct acute nerve injury during modeling, implying the disease may not have progressed to a stage inducing significant motor dysfunction. This result indicates that different types of neuropathic pain models may differ in their sensitivity to gait parameters. Future studies could further compare the performance of various neuropathic pain models in terms of motor function impairment and their responses to pharmacological interventions.

Chronic neuropathic pain is often accompanied by the release of abundant pro-inflammatory cytokines [46,47], and alleviating the pro-inflammatory environment at the injury site can mitigate chronic neuropathic pain [48]. Following peripheral nerve injury, microglia accumulate in the superficial dorsal horn within the termination zones of injured peripheral nerve fibers, promoting the release of various cytokines, primarily IL-1β, IL-6, and TNF-α. This enhances central neuronal sensitization, exacerbating pain [49]. While anti-inflammatory cytokines like IL-10 also exist, which can suppress inflammation to prevent excessive inflammatory responses that might harm the body, promote B-cell activation and expansion, and thus maintain immune homeostasis [50]. In this study, we found significantly elevated levels of IL-1β, TNF-α, and IL-6, along with reduced IL-10 expression, in tissue homogenates from diabetic neuropathic pain and postherpetic neuralgia model rats. After GeXIVA[1,2] treatment, levels of TNF-α, IL-1β, and IL-6 decreased, but IL-10 levels did not show a significant increase. This suggests that GeXIVA[1,2] may have a limited regulatory effect on anti-inflammatory cytokines. Notably, other α-conotoxins targeting α9α10 nAChR (e.g., Vc1.1, RgIA) exhibit similar regulatory profiles [51,52,53]. This suggests that α9α10 nAChR antagonists may restore a degree of inflammatory homeostasis by reducing pro-inflammatory cytokine levels, thereby exerting neuroprotective effects alongside analgesia.

Immunohistochemistry serves as a vital tool for studying cellular and molecular changes during nerve injury and repair, providing direct visualization of inflammatory cell infiltration [54]. In this study, we examined the injured sciatic nerve and adjacent muscle in diabetic neuropathic pain and postherpetic neuralgia rats using the markers CD2, CD68, and ChAT. Results showed significantly elevated expression of CD2 and CD68 in the model groups, indicating substantial infiltration of lymphocytes and macrophages in neuropathic pain models. These immune cells can persistently release pro-inflammatory cytokines like IL-1β, TNF-α, and IL-6 locally, thereby amplifying the inflammatory response and exacerbating nerve damage. After GeXIVA[1,2] treatment, the expression of CD2 and CD68 in the rat sciatic nerve significantly decreased, suggesting alleviation of inflammatory infiltration. The cumulative analgesic and nerve structure repair effects of GeXIVA[1,2] may stem from the restoration of immune balance, a result consistent with other studies on related α-conotoxins [55,56]. ChAT, a marker for cholinergic neurons, showed significantly reduced expression in diabetic neuropathic pain and postherpetic neuralgia model rats, indicating impaired cholinergic anti-inflammatory pathway function. After GeXIVA[1,2] treatment, we observed a marked increase in ChAT level, suggesting potential partial recovery of cholinergic anti-inflammatory pathway function.

However, our study has certain limitations. The experimental period was relatively short. Some patients with herpes zoster experience recurrence over 10–20 years [57], and diabetes with diabetic neuropathic pain potentially persisting throughout lifetime [58]. Therefore, chronic neuropathic pain patients often require long-term, even lifelong, treatment. Due to the limited observation period in this study, we were unable to comprehensively evaluate the longer-term sustained efficacy and potential impacts of GeXIVA[1,2].

## 4. Conclusions

In summary, this study evaluated the therapeutic effects of GeXIVA[1,2] in STZ-induced diabetic neuropathic pain and RTX-induced postherpetic neuralgia models. GeXIVA[1,2] effectively relieved neuropathic pain, improved motor coordination, reduced inflammation levels, and repaired damaged nerve structures. It represents a promising therapeutic agent for the treatment of both diabetic neuropathic pain and postherpetic neuralgia.

## 5. Materials and Methods

### 5.1. Drugs/Substances Used in the Study

Streptozocin (STZ), Tween 80, and citric acid were purchased from Macklin (Shanghai, China). Sodium citrate and gabapentin were purchased from Aladdin (Shanghai, China). Resiniferatoxin (RTX) was purchased from Beyanta Bio (Shanghai, China). ELISA kits for IL-1β, IL-6, TNF-α, and IL-10 were purchased from Kexing Trading Co., Ltd. (Shanghai, China). Rats were provided by the Experimental Animal Center of Guangxi Medical University (Nanning, China). GeXIVA[1,2] was synthesized and modified by our research group [19].

### 5.2. Animals

Adult male Sprague-Dawley (SD) rats, all aged six weeks and weighing 150–180 g, were used. Animal studies were conducted in accordance with guidelines established by the Institutional Animal Care Committee of Guangxi University (NO. GXU-2024-207). Animals were housed randomly in cages (six per cage) under a 12-h light/dark cycle at a constant temperature (24 ± 1 °C) and humidity (60 ± 5%), with free access to food (standard rodent diet) and water. Animals were acclimatized for one week prior to experiments. For all behavioral tests, the experimental environment was kept quiet, the same experimenter conducted the tests, and experiments were performed between 9:00 AM–12:00 PM and 3:00 PM–5:00 PM. All experiments involving animal behavior tests followed the double-blind principle.

### 5.3. Dose–Response Relationship

#### 5.3.1. Neuropathic Pain Model Establishment

Diabetic model: STZ was dissolved in a citrate buffer (pH 4.5, 0.1 M) to prepare a streptozocin solution (10 mg/mL). After a 16-h fast, rats were administered STZ (40 mg/kg) via a single daily intraperitoneal injection at the same time for 3 days. Blood samples were collected from the tail tip, and blood glucose levels were measured using a glucometer (Model 580, Yuwell, Danyang, China) 72 h later. All measurements were taken between 9:00 AM and 10:00 AM. Diabetic model rats were considered successfully established when their random blood glucose levels exceeded 16.7 mmol/L [59]. Rats did not receive insulin treatment throughout the experimental period.

Postherpetic neuralgia model: RTX (1 mg) was dissolved in a mixture of 2.5 mL of 10% Tween 80, 2.5 mL of 10% ethanol, and 20 mL of 80% saline to prepare a RTX solution (40 μg/mL). This solution was administered once via intraperitoneal injection at a dose of 200 μg/kg [60].

#### 5.3.2. Mechanical PWT Detection

Rats were placed in a plastic cage with a wire mesh bottom allowing full access to their paws. They were acclimated for 30 min until exploratory and grooming activities ceased. For the initial measurement, a 0.6 g Von Frey filament was applied vertically to the mid-plantar surface of the left hind paw. If the rat exhibited a clear withdrawal or licking response, a filament with a lower force was used in the subsequent measurement. If no response was observed, a filament with a higher force was used in the next trial. This process continued until six valid readings were obtained to calculate the baseline PWT. GeXIVA[1,2] was diluted with saline to target concentrations and administered via subcutaneous injection on the left thigh (0.1 mL/rat). Rats were randomly assigned to groups receiving different doses of GeXIVA[1,2] (5, 10, 15, 20 nmol) or a saline control group. For both diabetic and postherpetic neuralgia models, changes in PWT were measured at 0, 1, 2, 4, and 6 h after a single administration.

### 5.4. Evaluation of the Analgesic Effect of GeXIVA[1,2]

Diabetic neuropathic pain: Rats were randomly divided into 4 groups (*n* = 6 per group): (1) control group; (2) diabetic neuropathic pain group without treatment; (3) diabetic neuropathic pain + gabapentin treatment group; (4) diabetic neuropathic pain + GeXIVA[1,2] treatment group. Postherpetic neuralgia: Rats were randomly divided into 4 groups (*n* = 6 per group): (1) Control group; (2) Postherpetic neuralgia group without treatment; (3) Postherpetic neuralgia + Gabapentin treatment group; (4) Postherpetic neuralgia + GeXIVA[1,2] treatment group. Treatment starting from day 7 (Diabetic neuropathic pain group) or day 14 (Postherpetic neuralgia group) after modeling, GeXIVA[1,2] was administered subcutaneously at a dose of 15 nmol once daily for 21 consecutive days. The control and model groups received an equivalent volume of saline. The gabapentin treatment group received gabapentin orally (gavage) at a dose of 100 mg/kg (1 mL/kg). PWT was measured daily at 2 h post-administration.

### 5.5. Effect of GeXIVA[1,2] on Motor Function

#### 5.5.1. Rotarod Test

Motor coordination was assessed using a rat rotarod apparatus (Ugo Basile, Milan, Italy). Prior to testing, rats underwent a 3-day adaptation period to the testing environment. Rats were carefully placed on the rotating rod with their fore and hind paws facing away from the direction of rotation. During adaptation, a relatively slow speed (10 rpm) was set. Rats were immediately returned to the rod if they fell, with a training duration of 10 min and a 5-min rest between sessions. In the formal test, rats were placed on the rod with an accelerating protocol (from 4 rpm to 40 rpm). The latency to fall from the rod was measured to evaluate motor coordination and balance. Once the rats fell off, the test was terminated and they were not placed back on the rotating rod. Each rat was tested three times with a 10-min inter-trial interval, and the average of the three trials was calculated. Measurements were taken every 7 days.

#### 5.5.2. Gait Analysis

Gait analysis was performed using the DigiGait™ Imaging System (Mouse Specifics Inc., Framingham, MA, USA) to analyze changes in motor patterns of rats. Parameters were set as follows: treadmill speed 10 cm/s, inclination 0°. Rats were placed on the treadmill, and testing began when the animal was calm. A high-speed camera (150 frames/second) positioned beneath the treadmill recorded the rat’s movement (the chin and hips were marked black to enhance limb identification). Locomotion was recorded for 2 min, and a segment containing at least 2 complete gait cycles was selected for analysis. Unilateral hind paw-related DigiGait parameters (swing of stride; stance of stride; stride length; paw area) were selected for statistical analysis. Measurements were taken every 7 days.

### 5.6. Analysis of Inflammatory Cytokines in Injured Nerves and Perineural Tissues

After treatment and behavioral tests, animals were sacrificed. The left sciatic nerve and surrounding tissue were dissected, rinsed with cold PBS to remove residual blood, weighed, and homogenized mechanically in cold PBS (pH 7.2–7.4, 0.01 mol/L) at a 10% (*w*/*v*) ratio. The homogenate was centrifuged at 5000 rpm for 15 min, and the supernatant was collected for total protein concentration measurement. The expression levels of pro-inflammatory cytokines (IL-1β, IL-6, TNF-α) and the anti-inflammatory cytokine (IL-10) in the tissue homogenate supernatant were quantified using ELISA.

### 5.7. Infiltration of Immune Cells Around Injured Nerves

The extent of macrophage and lymphocyte infiltration around the sciatic nerve was quantitatively analyzed using biotin-streptavidin labeled immunohistochemistry (IHC). Paraffin sections were prepared from sciatic nerve and surrounding muscle tissue. After deparaffinization and PBS washes, sections were incubated in 0.3% H_2_O_2_ solution for 25 min. Following serum blocking, sections were incubated overnight at 4 °C with primary antibodies against CD2, CD68, and ChAT. Subsequently, sections were incubated at room temperature for 50 min with corresponding horseradish peroxidase (HRP)-conjugated secondary antibodies. DAB chromogen was applied for color development. After counterstaining nuclei, dehydration, and mounting, immunostaining was observed and imaged using a camera (Nikon D90, Nikon Corporation, Tokyo, Japan) attached to an optical microscope (Eclipse LV100NDA LED, Nikon Corporation, Tokyo, Japan). Quantitative analysis of the sections was performed using ImageJ (1.54) software (National Institutes of Health, Bethesda, MD, USA).

### 5.8. Pathological Analysis of Sciatic Nerve Fiber Structure

Experimental rats were sacrificed after treatment, and a fresh 1 mm^3^ piece of the left sciatic nerve was harvested within 1–3 min. After double fixation with glutaraldehyde and osmium tetroxide, the tissue was dehydrated through a graded ethanol series, embedded in epoxy resin, and ultra-thin sections were prepared. Sections were double-stained with uranyl acetate and lead citrate. Ultrastructural changes in the nerves were observed using a transmission electron microscope (HT7800/HT7700, HITACHI, Hitachi, Japan). Myelin sheath thickness was quantitatively analyzed, and G-ratios (axon diameter/myelinated fiber diameter) were calculated using ImageJ software [61].

### 5.9. Statistical Analysis

All results are presented as mean ± standard deviation (SD). Statistical analysis was performed using GraphPad Prism 10 (San Diego, CA, USA). For comparisons involving more than two groups, one-way analysis of variance (ANOVA) was conducted, followed by post hoc pairwise comparisons using unpaired *t*-tests with Bonferroni correction for multiple comparisons. For comparisons between two groups, unpaired *t*-tests were used. A *p*-value of less than 0.05 was considered statistically significant.

## Data Availability

The original contributions presented in this study are included in the article/Supplementary Materials. Further inquiries can be directed to the corresponding authors.

## Supplementary Materials

The following supporting information can be downloaded at: https://www.mdpi.com/article/10.3390/toxins18060249/s1, Figure S1: Random blood glucose levels after STZ injections; Figure S2: Weight of diabetic neuropathic pain rats (A) and postherpetic neuralgia rats (B) during treatment; Figure S3: Standard curve for measuring inflammatory cytokines.

## Abbreviations

The following abbreviations are used in this manuscript:

TEM	Transmission electron microscope
CIPN	Chemotherapy-induced peripheral neuropathy
SNI	Spared nerve injury
CCI	Chronic constriction injury
STZ	Streptozocin
RTX	Resiniferatoxin
PWT	Paw withdrawal threshold
AUC	Area under the curve
