# Supplementary material for: Analgesic and Neurorestorative Effects of αO-Conotoxin GeXIVA[1,2] in Diabetic Neuropathic Pain and Postherpetic Neuralgia
Source: Toxins (Basel). 2026 May 29;18(6):249. doi: 10.3390/toxins18060249 (PMC13308345; doi:10.3390/toxins18060249)
Supplement: Supplementary file 1 [file toxins-18-00249-s001.zip › toxins-4233722-supplementary.pdf]

toxins

MDPI

Supplementary Information

Analgesic and Neurorestorative Effects of αO-conotoxin GeXIVA[1,2] in Diabetic Neuropathic Pain and Postherpetic Neuralgia

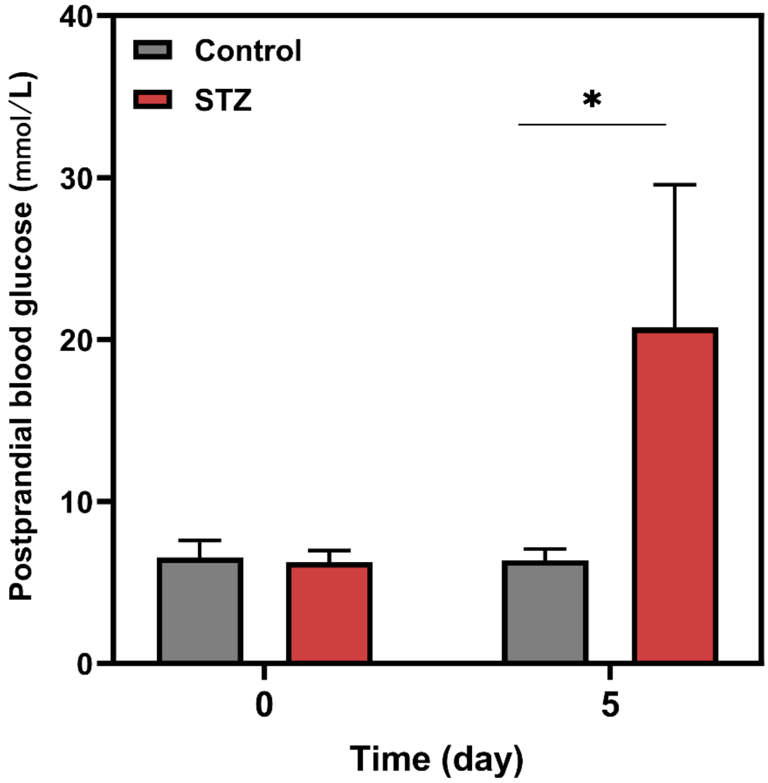

Figure S1. Random blood glucose levels after STZ injections.

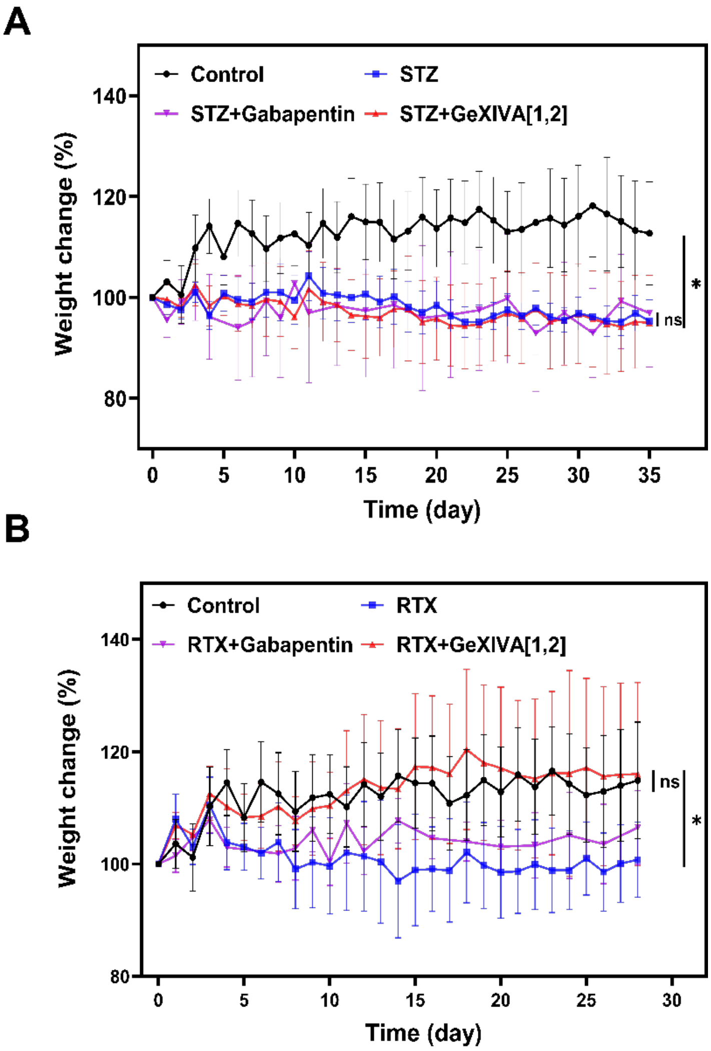

Figure S2. Weight of DNP rats (A) and PHN rats (B) during treatment.

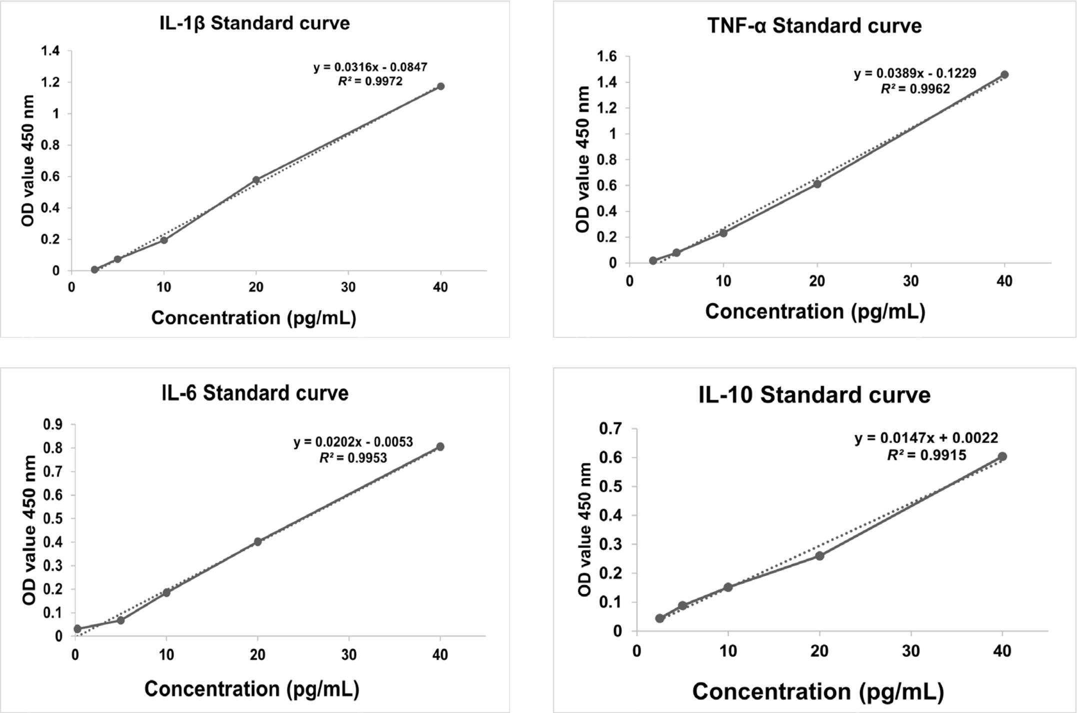

Figure S3. Standard curve for measuring inflammatory cytokines.

In the statistical analysis of this study, a one-way ANOVA was performed using GraphPad Prism (10) software, followed by post hoc pairwise comparisons using unpaired t-tests with Bonferroni correction for multiple comparisons. The complete statistical results, including the F values, degrees of freedom, and p values from the ANOVA, as well as the t values, degrees of freedom, and p values from the post hoc unpaired t-tests, are presented in tabular form below:

Figure 1B

ANOVA: $F(4, 25) = 57.46$ $p<0.0001$

Bonferonni's correction	p values	t values	degrees of freedoms
0 vs 5	0.7631	1.849	25
0 vs 10	0.0001	5.472	25
0 vs 15	<0.0001	11.68	25
0 vs 20	<0.0001	11.40	25
5 vs 10	0.0129	3.623	25
5 vs 15	<0.0001	9.835	25
5 vs 20	<0.0001	9.549	25
10 vs 15	<0.0001	6.212	25
10 vs 20	<0.0001	5.926	25
15 vs 20	>0.9999	0.2856	25

Figure 1D

ANOVA: $F(4, 25) = 54.67$ $p<0.0001$

Bonferonni's correction	p values	t values	degrees of freedoms
0 vs 5	>0.9999	1.213	25
0 vs 10	0.9425	1.739	25
0 vs 15	<0.0001	11.27	25
0 vs 20	<0.0001	9.512	25
5 vs 10	>0.9999	0.5268	25
5 vs 15	<0.0001	10.06	25
5 vs 20	<0.0001	8.299	25
10 vs 15	<0.0001	9.532	25
10 vs 20	<0.0001	7.772	25
15 vs 20	0.9062	1.760	25

Figure 2C

ANOVA: F (3, 20) = 71.25 p<0.0001

Bonferonni's correction	p values	t values	degrees of freedoms
Control vs STZ	<0.0001	13.30	20
Control vs STZ + Gabapentin(100 mg/kg, PO)	0.0003	5.099	20
Control vs STZ + GeXIVA[1,2](15 nmol, SC)	>0.9999	1.392	20
STZ vs STZ + Gabapentin(100 mg/kg, PO)	<0.0001	8.199	20
STZ vs STZ + GeXIVA[1,2](15 nmol, SC)	<0.0001	11.91	20
STZ + Gabapentin(100 mg/kg, PO) vs STZ + GeXIVA[1,2](15 nmol, SC)	0.0084	3.707	20

Figure 3C

ANOVA: F (3, 20) = 44.05 p<0.0001

Bonferonni's correction	p values	t values	degrees of freedoms
Control vs RTX	<0.0001	10.66	20
Control vs RTX + Gabapentin(100 mg/kg, PO)	0.0003	5.155	20
Control vs RTX + GeXIVA[1,2](15 nmol, SC)	0.5542	1.768	20
RTX vs RTX + Gabapentin(100 mg/kg, PO)	0.0001	5.507	20
RTX vs RTX + GeXIVA[1,2](15 nmol, SC)	<0.0001	8.894	20
RTX + Gabapentin(100 mg/kg, PO) vs RTX + GeXIVA[1,2] (15 nmol, SC)	0.0176	3.387	20

Figure 5A

ANOVA: F (3, 80) = 19.55 p<0.0001

The statistical results for Day 0 (before the start of treatment) are as follows:

Bonferonni's correction	p values	t values	degrees of freedoms
Control vs RTX	0.0004	4.185	80.00
Control vs RTX + Gabapentin	0.0018	3.786	80.00
Control vs RTX + GeXIVA[1,2]	0.0035	3.584	80.00
RTX vs RTX + Gabapentin	>0.9999	0.3989	80.00
RTX vs RTX + GeXIVA[1,2]	>0.9999	0.6012	80.00
RTX + Gabapentin vs RTX + GeXIVA[1,2]	>0.9999	0.2023	80.00

The statistical results for Day 21 (at the end of treatment) are as follows:

Bonferonni's correction	p values	t values	degrees of freedoms
Control vs RTX	0.0125	3.183	80.00
Control vs RTX + Gabapentin	>0.9999	0.5234	80.00
Control vs RTX + GeXIVA[1,2]	>0.9999	0.3998	80.00
RTX vs RTX + Gabapentin	0.0567	2.660	80.00
RTX vs RTX + GeXIVA[1,2]	0.0403	2.783	80.00
RTX + Gabapentin vs RTX + GeXIVA[1,2]	>0.9999	0.1235	80.00

Figure 5B, C

ANOVA: F (3, 80) = 14.25 p<0.0001

The statistical results for Day 21 (at the end of treatment) are as follows:

Bonferonni's correction	p values	t values	degrees of free-doms
Control vs RTX	0.0172	3.077	80.00
Control vs RTX + Gabapentin	0.0481	2.719	80.00
Control vs RTX + GeXIVA[1,2]	>0.9999	0.01570	80.00
RTX vs RTX + Gabapentin	>0.9999	0.3571	80.00
RTX vs RTX + GeXIVA[1,2]	0.0164	3.092	80.00
RTX + Gabapentin vs RTX + GeXIVA[1,2]	0.0461	2.735	80.00

Figure 5D

ANOVA: F (3, 80) = 3.550 p<0.0001

The statistical results for Day 21 (at the end of treatment) are as follows:

Bonferonni's correction	p values	t values	degrees of free-doms
Control vs RTX	0.9278	1.437	80.00
Control vs RTX + Gabapentin	0.4269	1.829	80.00
Control vs RTX + GeXIVA[1,2]	>0.9999	0.6042	80.00
RTX vs RTX + Gabapentin	>0.9999	0.3919	80.00
RTX vs RTX + GeXIVA[1,2]	>0.9999	0.8328	80.00
RTX + Gabapentin vs RTX + GeXIVA[1,2]	>0.9999	1.225	80.00

Figure 5E

ANOVA: $F (3, 80) = 20.24$ $p<0.0001$

The statistical results for Day 21 (at the end of treatment) are as follows:

Bonferonni's correction	p values	t values	degrees of free-doms
Control vs RTX	0.1854	2.197	80.00
Control vs RTX + Gabapentin	0.0004	4.195	80.00
Control vs RTX + GeXIVA[1,2]	0.1918	2.183	80.00
RTX vs RTX + Gabapentin	0.2948	1.998	80.00
RTX vs RTX + GeXIVA[1,2]	>0.9999	0.01424	80.00
RTX + Gabapentin vs RTX + GeXIVA[1,2]	0.2855	2.012	80.00

Figure 7B

ANOVA: F (2, 24) = 15.27 p<0.0001

Bonferonni's correction	p values	t values	degrees of free-doms
Control vs STZ	<0.0001	5.523	24
Control vs STZ + GeXIVA[1,2]	0.0476	2.595	24
STZ vs STZ + GeXIVA[1,2]	0.0221	2.928	24

Figure 7C

ANOVA: F (2, 24) = 18.44 p<0.0001

Bonferonni's correction	p values	t values	degrees of free-doms
Control vs STZ	<0.0001	6.015	24
Control vs STZ + GeXIVA[1,2]	0.0958	2.278	24
STZ vs STZ + GeXIVA[1,2]	0.0031	3.737	24

Figure 7D

ANOVA: F (2, 24) = 31.45 p<0.0001

Bonferonni's correction	p values	t values	degrees of free-doms
Control vs STZ	>0.9999	0.5013	24
Control vs STZ + GeXIVA[1,2]	<0.0001	6.604	24
STZ vs STZ + GeXIVA[1,2]	<0.0001	7.106	24

Figure 7E

ANOVA: F (2, 24) = 25.08 p<0.0001

Bonferonni's correction	p values	t values	degrees of free-doms
Control vs STZ	<0.0001	5.408	24
Control vs STZ + GeXIVA[1,2]	0.6638	1.256	24
STZ vs STZ + GeXIVA[1,2]	<0.0001	6.664	24

Figure 7F

ANOVA: F (2, 24) = 60.39 p<0.0001

Bonferonni's correction	p values	t values	degrees of free-doms
Control vs STZ	<0.0001	8.989	24
Control vs STZ + GeXIVA[1,2]	>0.9999	0.9809	24
STZ vs STZ + GeXIVA[1,2]	<0.0001	9.970	24

Figure 7G

ANOVA: F (2, 24) = 5.576 p<0.0001

Bonferonni's correction	p values	t values	degrees of free-doms
Control vs STZ	0.8814	1.073	24
Control vs STZ + GeXIVA[1,2]	0.1126	2.202	24
STZ vs STZ + GeXIVA[1,2]	0.0096	3.275	24

Figure 8B

ANOVA: F (2, 24) = 21.21 p<0.0001

Bonferonni's correction	p values	t values	degrees of free-doms
Control vs RTX	0.0003	4.694	24
Control vs RTX + GeXIVA[1,2]	0.3941	1.562	24
RTX vs RTX + GeXIVA[1,2]	<0.0001	6.256	24

Figure 8C

ANOVA: F (2, 24) = 18.40 p<0.0001

Bonferonni's correction	p values	t values	degrees of free-doms
Control vs RTX	0.0005	4.425	24
Control vs RTX + GeXIVA[1,2]	0.5388	1.382	24
RTX vs RTX + GeXIVA[1,2]	<0.0001	5.807	24

Figure 8D

ANOVA: F (2, 24) = 13.01 p<0.0001

Bonferonni's correction	p values	t values	degrees of free-doms
Control vs RTX	0.0184	3.004	24
Control vs RTX + GeXIVA[1,2]	0.1487	2.068	24
RTX vs RTX + GeXIVA[1,2]	0.0001	5.072	24

Figure 8E

ANOVA: F (2, 24) = 22.08   p<0.0001

Bonferonni's correction	p values	t values	degrees of freedoms
Control vs RTX	<0.0001	5.606	24
Control vs RTX + GeXIVA[1,2]	>0.9999	0.2883	24
RTX vs RTX + GeXIVA[1,2]	<0.0001	5.894	24

Figure 8F

ANOVA: F (2, 24) = 30.76   p<0.0001

Bonferonni's correction	p values	t values	degrees of freedoms
Control vs RTX	<0.0001	7.225	24
Control vs RTX + GeXIVA[1,2]	>0.9999	0.9701	24
RTX vs RTX + GeXIVA[1,2]	<0.0001	6.255	24

Figure 8G

ANOVA: F (2, 24) = 9.124   p<0.0001

Bonferonni's correction	p values	t values	degrees of freedoms
Control vs RTX	0.5559	1.364	24
Control vs RTX + GeXIVA[1,2]	0.0282	2.824	24
RTX vs RTX + GeXIVA[1,2]	0.0010	4.188	24

Figure 9C

ANOVA: F (2, 117) = 4.483 p=0.0133

Bonferonni's correction	p values	t values	degrees of free-doms
Control vs STZ	0.0287	2.635	117
Control vs STZ + GeXIVA[1,2]	>0.9999	0.08499	117
STZ vs STZ + GeXIVA[1,2]	0.0362	2.550	117

Figure 9D

ANOVA: F (2, 117) = 67.21 p<0.0001

Bonferonni's correction	p values	t values	degrees of free-doms
Control vs STZ	<0.0001	11.22	117
Control vs STZ + GeXIVA[1,2]	0.0079	3.073	117
STZ vs STZ + GeXIVA[1,2]	<0.0001	8.145	117

Figure 9E

ANOVA: F (2, 87) = 18.93 p<0.0001

Bonferonni's correction	p values	t values	degrees of free-doms
Control vs RTX	<0.0001	6.152	87
Control vs RTX + GeXIVA[1,2]	0.0073	3.122	87
RTX vs RTX + GeXIVA[1,2]	0.0097	3.030	87

Figure 9F

ANOVA: $F (2, 87) = 24.67$ $p<0.0001$

Bonferonni's correction	p values	t values	degrees of free-doms
Control vs RTX	<0.0001	7.020	87
Control vs RTX + GeXIVA[1,2]	0.0040	3.315	87
RTX vs RTX + GeXIVA[1,2]	0.0011	3.706	87
